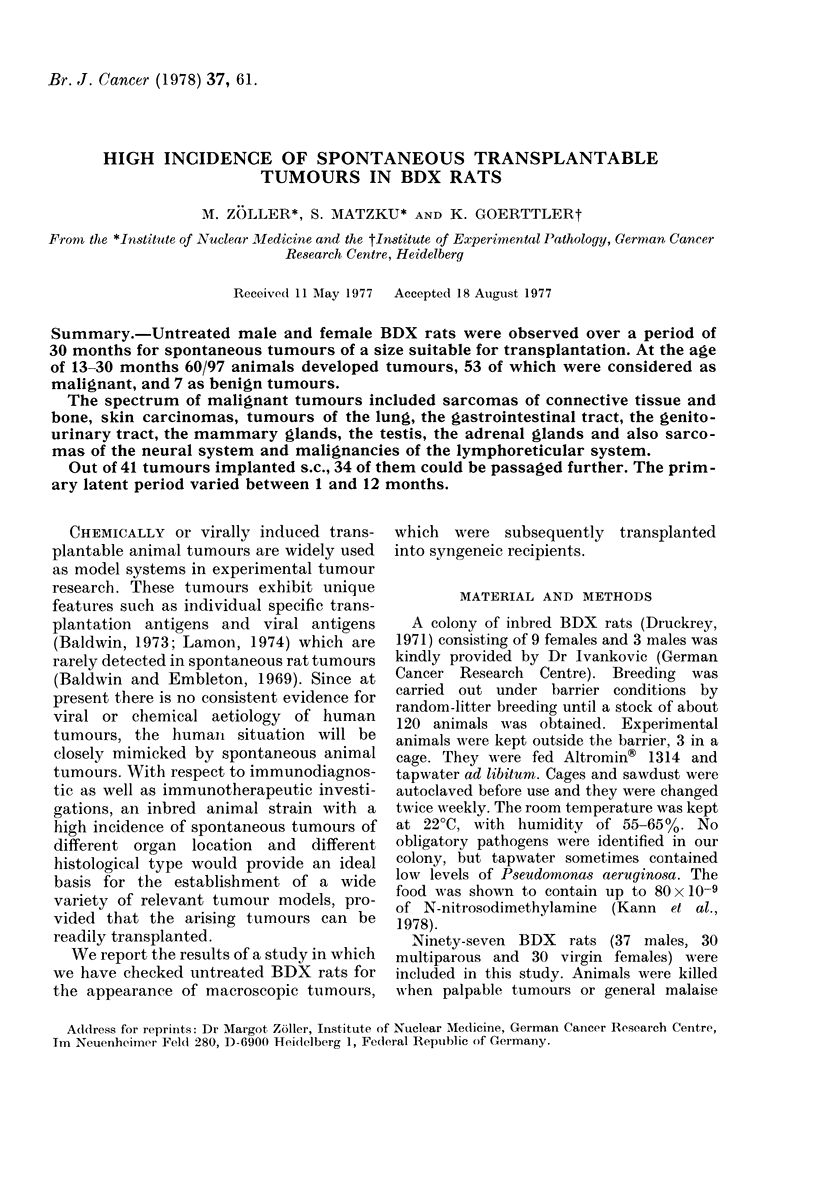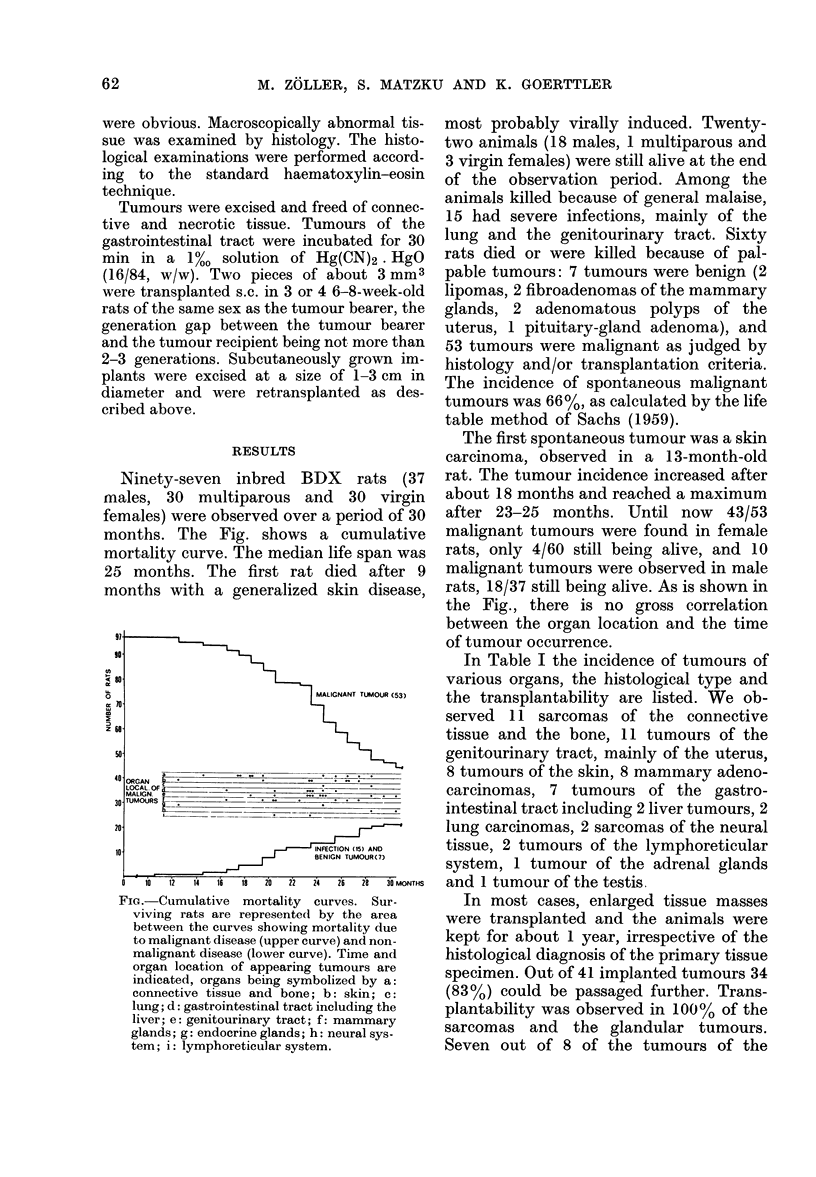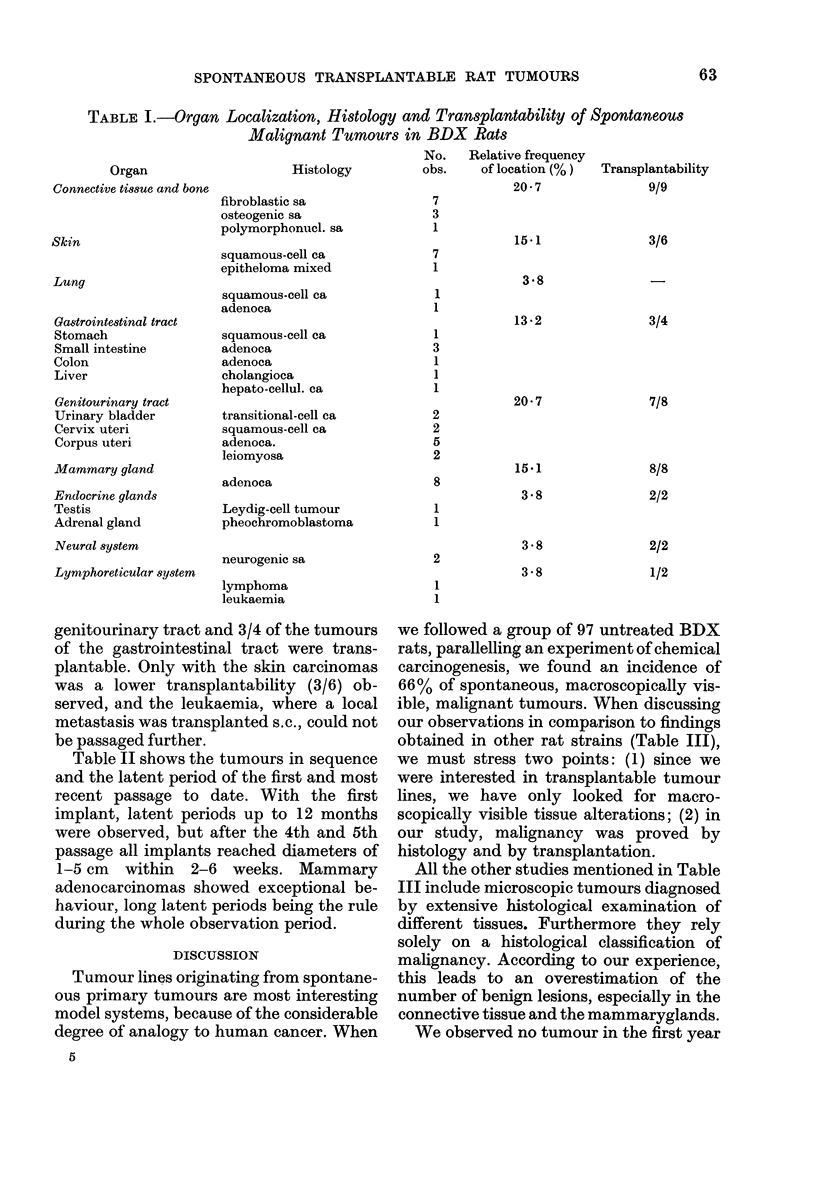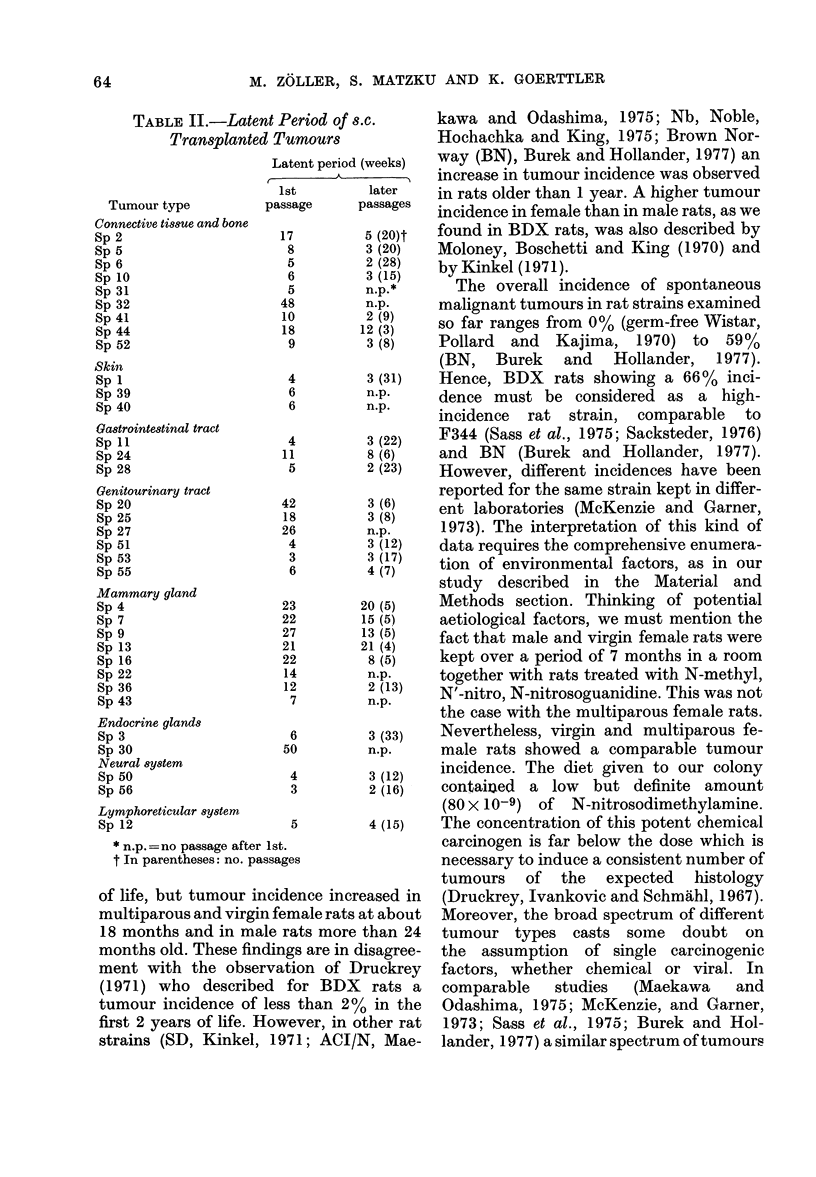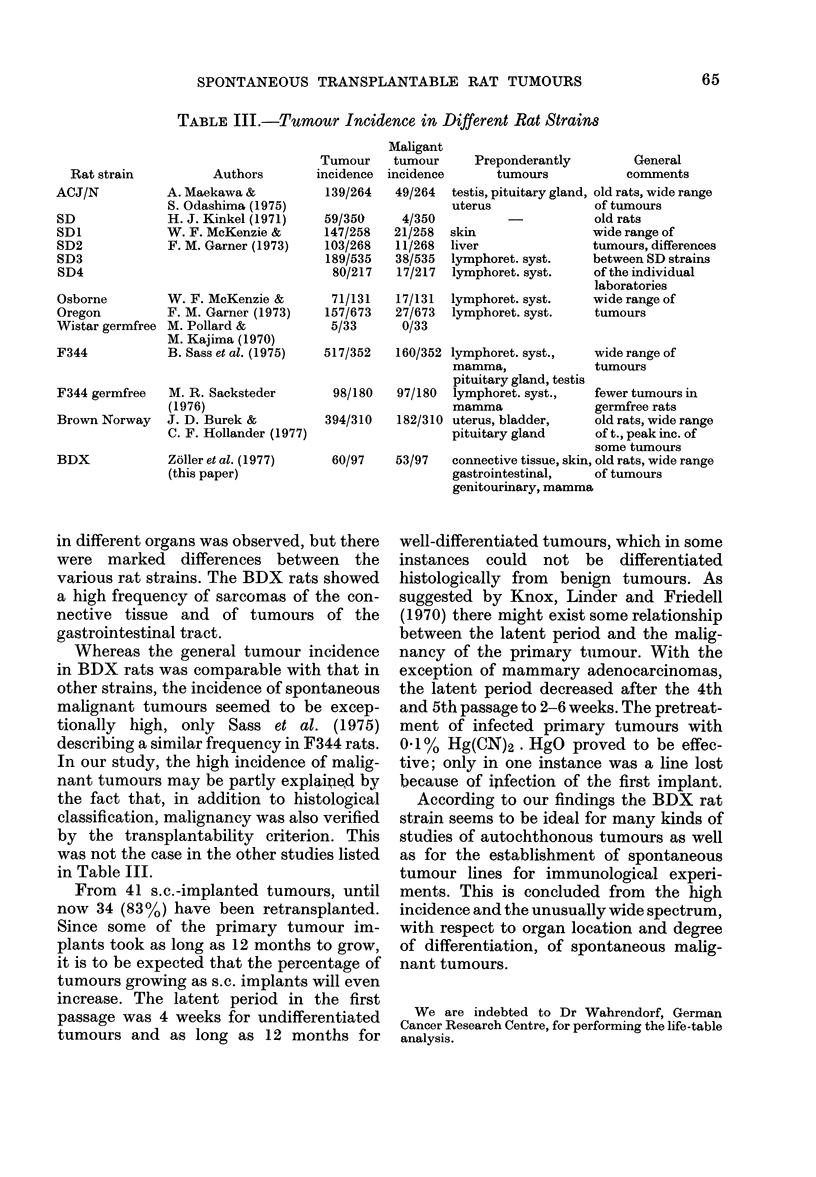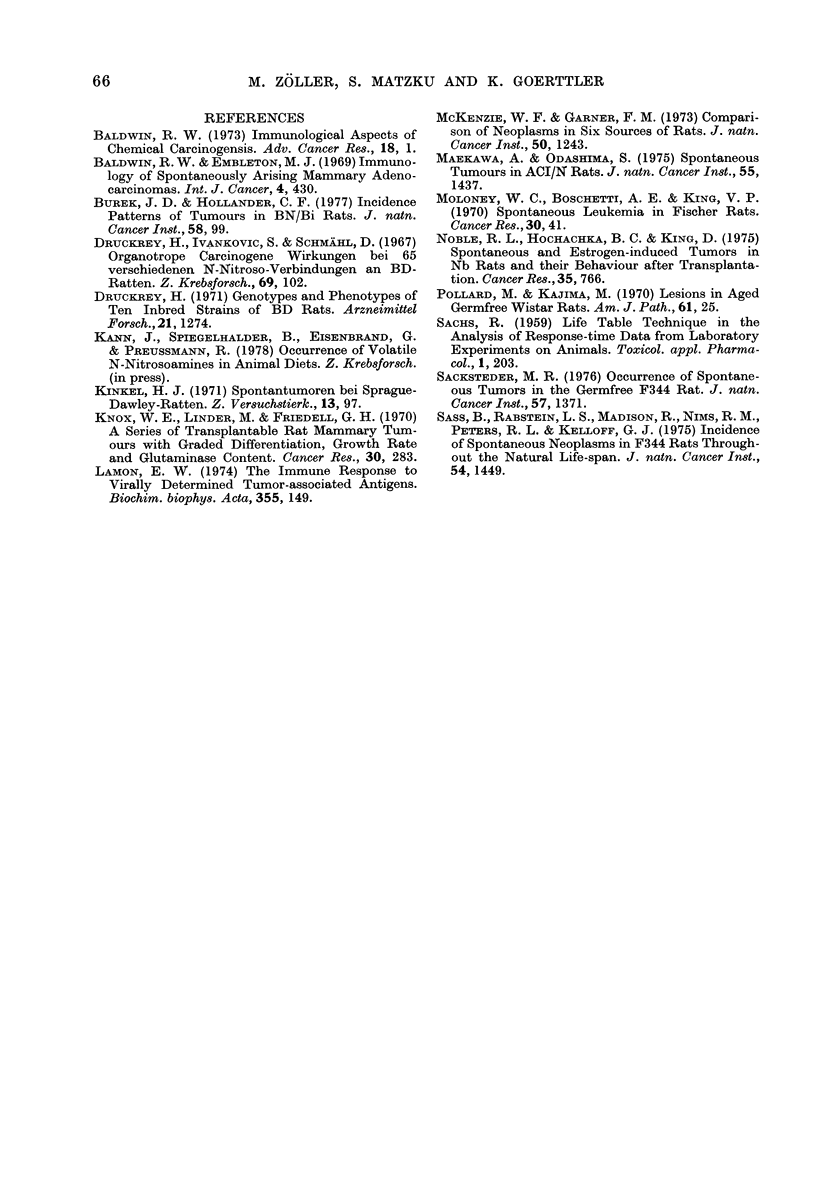# High incidence of spontaneous transplantable tumours in BDX rats.

**DOI:** 10.1038/bjc.1978.9

**Published:** 1978-01

**Authors:** M. Zöller, S. Matzku, K. Goerttler

## Abstract

Untreated male and female BDX rats were observed over a period of 30 months for spontaneous tumours of a size suitable for transplantation. At the age of 13-30 months 60/97 animals developed tumours, 53 of which were considered as malignant, and 7 as benign tumours. The spectrum of malignant tumours included sarcomas of connective tissue and bone, skin carcinomas, tumours of the lung, the gastrointestinal tract, the genito-urinary tract, the mammary glands, the testis, the adrenal glands and also sarcomas of the neural system and malignancies of the lymphoreticular system. Out of 41 tumours implanted s.c., 34 of them could be passaged further. The primary latent period varied between 1 and 12 months.


*Br. J. Cancer* (1978) **37**, 61.

### HIGH INCIDENCE OF SPONTANEOUS TRANSPLANTABLE TUMOURS IN BDX RATS

M. ZÖLLER*, S. MATZKU* AND K. GOERTTLER†

*From the *Institute of Nuclear Medicine and the †Institute of Experimental Pathology, German Cancer Research Centre, Heidelberg*

Received 11 May 1977 Accepted 18 August 1977

**Summary.—Untreated male and female BDX rats were observed over a period of 30 months for spontaneous tumours of a size suitable for transplantation. At the age of 13–30 months 60/97 animals developed tumours, 53 of which were considered as malignant, and 7 as benign tumours.**

**The spectrum of malignant tumours included sarcomas of connective tissue and bone, skin carcinomas, tumours of the lung, the gastrointestinal tract, the genito-urinary tract, the mammary glands, the testis, the adrenal glands and also sarcomas of the neural system and malignancies of the lymphoreticular system.**

**Out of 41 tumours implanted s.c., 34 of them could be passaged further. The primary latent period varied between 1 and 12 months.**

CHEMICALLY or virally induced transplantable animal tumours are widely used as model systems in experimental tumour research. These tumours exhibit unique features such as individual specific transplantation antigens and viral antigens (Baldwin, 1973; Lamon, 1974) which are rarely detected in spontaneous rat tumours (Baldwin and Embleton, 1969). Since at present there is no consistent evidence for viral or chemical aetiology of human tumours, the human situation will be closely mimicked by spontaneous animal tumours. With respect to immunodiagnostic as well as immunotherapeutic investigations, an inbred animal strain with a high incidence of spontaneous tumours of different organ location and different histological type would provide an ideal basis for the establishment of a wide variety of relevant tumour models, provided that the arising tumours can be readily transplanted.

We report the results of a study in which we have checked untreated BDX rats for the appearance of macroscopic tumours, which were subsequently transplanted into syngeneic recipients.

### MATERIAL AND METHODS

A colony of inbred BDX rats (Druckrey, 1971) consisting of 9 females and 3 males was kindly provided by Dr Ivankovic (German Cancer Research Centre). Breeding was carried out under barrier conditions by random-litter breeding until a stock of about 120 animals was obtained. Experimental animals were kept outside the barrier, 3 in a cage. They were fed Altromin® 1314 and tapwater *ad libitum*. Cages and sawdust were autoclaved before use and they were changed twice weekly. The room temperature was kept at 22°C, with humidity of 55–65%. No obligatory pathogens were identified in our colony, but tapwater sometimes contained low levels of *Pseudomonas aeruginosa*. The food was shown to contain up to $80 \times 10^{-9}$ of N-nitrosodimethylamine (Kann *et al.*, 1978).

Ninety-seven BDX rats (37 males, 30 multiparous and 30 virgin females) were included in this study. Animals were killed when palpable tumours or general malaise

Address for reprints: Dr Margot Zöller, Institute of Nuclear Medicine, German Cancer Research Centre, Im Neuenheimer Feld 280, D-6900 Heidelberg 1, Federal Republic of Germany.

were obvious. Macroscopically abnormal tissue was examined by histology. The histological examinations were performed according to the standard haematoxylin–eosin technique.

Tumours were excised and freed of connective and necrotic tissue. Tumours of the gastrointestinal tract were incubated for 30 min in a 1‰ solution of $Hg(CN)_2 . HgO$ (16/84, w/w). Two pieces of about 3 mm³ were transplanted s.c. in 3 or 4 6–8-week-old rats of the same sex as the tumour bearer, the generation gap between the tumour bearer and the tumour recipient being not more than 2–3 generations. Subcutaneously grown implants were excised at a size of 1–3 cm in diameter and were retransplanted as described above.

### RESULTS

Ninety-seven inbred BDX rats (37 males, 30 multiparous and 30 virgin females) were observed over a period of 30 months. The Fig. shows a cumulative mortality curve. The median life span was 25 months. The first rat died after 9 months with a generalized skin disease,

Fig.—Cumulative mortality curves. Surviving rats are represented by the area between the curves showing mortality due to malignant disease (upper curve) and non-malignant disease (lower curve). Time and organ location of appearing tumours are indicated, organs being symbolized by a: connective tissue and bone; b: skin; c: lung; d: gastrointestinal tract including the liver; e: genitourinary tract; f: mammary glands; g: endocrine glands; h: neural system; i: lymphoreticular system.

most probably virally induced. Twenty-two animals (18 males, 1 multiparous and 3 virgin females) were still alive at the end of the observation period. Among the animals killed because of general malaise, 15 had severe infections, mainly of the lung and the genitourinary tract. Sixty rats died or were killed because of palpable tumours: 7 tumours were benign (2 lipomas, 2 fibroadenomas of the mammary glands, 2 adenomatous polyps of the uterus, 1 pituitary-gland adenoma), and 53 tumours were malignant as judged by histology and/or transplantation criteria. The incidence of spontaneous malignant tumours was 66%, as calculated by the life table method of Sachs (1959).

The first spontaneous tumour was a skin carcinoma, observed in a 13-month-old rat. The tumour incidence increased after about 18 months and reached a maximum after 23–25 months. Until now 43/53 malignant tumours were found in female rats, only 4/60 still being alive, and 10 malignant tumours were observed in male rats, 18/37 still being alive. As is shown in the Fig., there is no gross correlation between the organ location and the time of tumour occurrence.

In Table I the incidence of tumours of various organs, the histological type and the transplantability are listed. We observed 11 sarcomas of the connective tissue and the bone, 11 tumours of the genitourinary tract, mainly of the uterus, 8 tumours of the skin, 8 mammary adenocarcinomas, 7 tumours of the gastrointestinal tract including 2 liver tumours, 2 lung carcinomas, 2 sarcomas of the neural tissue, 2 tumours of the lymphoreticular system, 1 tumour of the adrenal glands and 1 tumour of the testis.

In most cases, enlarged tissue masses were transplanted and the animals were kept for about 1 year, irrespective of the histological diagnosis of the primary tissue specimen. Out of 41 implanted tumours 34 (83%) could be passaged further. Transplantability was observed in 100% of the sarcomas and the glandular tumours. Seven out of 8 of the tumours of the

TABLE I.—*Organ Localization, Histology and Transplantability of Spontaneous Malignant Tumours in BDX Rats*

| Organ | Histology | No. obs. | Relative frequency of location (%) | Transplantability |
|---|---|---|---|---|
| *Connective tissue and bone* | | | 20·7 | 9/9 |
| | fibroblastic sa | 7 | | |
| | osteogenic sa | 3 | | |
| | polymorphonucl. sa | 1 | | |
| *Skin* | | | 15·1 | 3/6 |
| | squamous-cell ca | 7 | | |
| | epitheloma mixed | 1 | | |
| *Lung* | | | 3·8 | — |
| | squamous-cell ca | 1 | | |
| | adenoca | 1 | | |
| *Gastrointestinal tract* | | | 13·2 | 3/4 |
| Stomach | squamous-cell ca | 1 | | |
| Small intestine | adenoca | 3 | | |
| Colon | adenoca | 1 | | |
| Liver | cholangioca | 1 | | |
| | hepato-cellul. ca | 1 | | |
| *Genitourinary tract* | | | 20·7 | 7/8 |
| Urinary bladder | transitional-cell ca | 2 | | |
| Cervix uteri | squamous-cell ca | 2 | | |
| Corpus uteri | adenoca. | 5 | | |
| | leiomyosa | 2 | | |
| *Mammary gland* | | | 15·1 | 8/8 |
| | adenoca | 8 | | |
| *Endocrine glands* | | | 3·8 | 2/2 |
| Testis | Leydig-cell tumour | 1 | | |
| Adrenal gland | pheochromoblastoma | 1 | | |
| *Neural system* | | | 3·8 | 2/2 |
| | neurogenic sa | 2 | | |
| *Lymphoreticular system* | | | 3·8 | 1/2 |
| | lymphoma | 1 | | |
| | leukaemia | 1 | | |

genitourinary tract and 3/4 of the tumours of the gastrointestinal tract were transplantable. Only with the skin carcinomas was a lower transplantability (3/6) observed, and the leukaemia, where a local metastasis was transplanted s.c., could not be passaged further.

Table II shows the tumours in sequence and the latent period of the first and most recent passage to date. With the first implant, latent periods up to 12 months were observed, but after the 4th and 5th passage all implants reached diameters of 1–5 cm within 2–6 weeks. Mammary adenocarcinomas showed exceptional behaviour, long latent periods being the rule during the whole observation period.

### DISCUSSION

Tumour lines originating from spontaneous primary tumours are most interesting model systems, because of the considerable degree of analogy to human cancer. When we followed a group of 97 untreated BDX rats, parallelling an experiment of chemical carcinogenesis, we found an incidence of 66% of spontaneous, macroscopically visible, malignant tumours. When discussing our observations in comparison to findings obtained in other rat strains (Table III), we must stress two points: (1) since we were interested in transplantable tumour lines, we have only looked for macroscopically visible tissue alterations; (2) in our study, malignancy was proved by histology and by transplantation.

All the other studies mentioned in Table III include microscopic tumours diagnosed by extensive histological examination of different tissues. Furthermore they rely solely on a histological classification of malignancy. According to our experience, this leads to an overestimation of the number of benign lesions, especially in the connective tissue and the mammaryglands.

We observed no tumour in the first year

TABLE II.—*Latent Period of s.c. Transplanted Tumours*

| Tumour type | Latent period (weeks) 1st passage | later passages |
|---|---|---|
| *Connective tissue and bone* | | |
| Sp 2 | 17 | 5 (20)† |
| Sp 5 | 8 | 3 (20) |
| Sp 6 | 5 | 2 (28) |
| Sp 10 | 6 | 3 (15) |
| Sp 31 | 5 | n.p.* |
| Sp 32 | 48 | n.p. |
| Sp 41 | 10 | 2 (9) |
| Sp 44 | 18 | 12 (3) |
| Sp 52 | 9 | 3 (8) |
| *Skin* | | |
| Sp 1 | 4 | 3 (31) |
| Sp 39 | 6 | n.p. |
| Sp 40 | 6 | n.p. |
| *Gastrointestinal tract* | | |
| Sp 11 | 4 | 3 (22) |
| Sp 24 | 11 | 8 (6) |
| Sp 28 | 5 | 2 (23) |
| *Genitourinary tract* | | |
| Sp 20 | 42 | 3 (6) |
| Sp 25 | 18 | 3 (8) |
| Sp 27 | 26 | n.p. |
| Sp 51 | 4 | 3 (12) |
| Sp 53 | 3 | 3 (17) |
| Sp 55 | 6 | 4 (7) |
| *Mammary gland* | | |
| Sp 4 | 23 | 20 (5) |
| Sp 7 | 22 | 15 (5) |
| Sp 9 | 27 | 13 (5) |
| Sp 13 | 21 | 21 (4) |
| Sp 16 | 22 | 8 (5) |
| Sp 22 | 14 | n.p. |
| Sp 36 | 12 | 2 (13) |
| Sp 43 | 7 | n.p. |
| *Endocrine glands* | | |
| Sp 3 | 6 | 3 (33) |
| Sp 30 | 50 | n.p. |
| *Neural system* | | |
| Sp 50 | 4 | 3 (12) |
| Sp 56 | 3 | 2 (16) |
| *Lymphoreticular system* | | |
| Sp 12 | 5 | 4 (15) |

* n.p. = no passage after 1st.
† In parentheses: no. passages of life, but tumour incidence increased in multiparous and virgin female rats at about 18 months and in male rats more than 24 months old. These findings are in disagreement with the observation of Druckrey (1971) who described for BDX rats a tumour incidence of less than 2% in the first 2 years of life. However, in other rat strains (SD, Kinkel, 1971; ACI/N, Maekawa and Odashima, 1975; Nb, Noble, Hochachka and King, 1975; Brown Norway (BN), Burek and Hollander, 1977) an increase in tumour incidence was observed in rats older than 1 year. A higher tumour incidence in female than in male rats, as we found in BDX rats, was also described by Moloney, Boschetti and King (1970) and by Kinkel (1971).

The overall incidence of spontaneous malignant tumours in rat strains examined so far ranges from 0% (germ-free Wistar, Pollard and Kajima, 1970) to 59% (BN, Burek and Hollander, 1977). Hence, BDX rats showing a 66% incidence must be considered as a high-incidence rat strain, comparable to F344 (Sass *et al.*, 1975; Sacksteder, 1976) and BN (Burek and Hollander, 1977). However, different incidences have been reported for the same strain kept in different laboratories (McKenzie and Garner, 1973). The interpretation of this kind of data requires the comprehensive enumeration of environmental factors, as in our study described in the Material and Methods section. Thinking of potential aetiological factors, we must mention the fact that male and virgin female rats were kept over a period of 7 months in a room together with rats treated with N-methyl, N′-nitro, N-nitrosoguanidine. This was not the case with the multiparous female rats. Nevertheless, virgin and multiparous female rats showed a comparable tumour incidence. The diet given to our colony contained a low but definite amount ($80 \times 10^{-9}$) of N-nitrosodimethylamine. The concentration of this potent chemical carcinogen is far below the dose which is necessary to induce a consistent number of tumours of the expected histology (Druckrey, Ivankovic and Schmähl, 1967). Moreover, the broad spectrum of different tumour types casts some doubt on the assumption of single carcinogenic factors, whether chemical or viral. In comparable studies (Maekawa and Odashima, 1975; McKenzie, and Garner, 1973; Sass *et al.*, 1975; Burek and Hollander, 1977) a similar spectrum of tumours

TABLE III.—*Tumour Incidence in Different Rat Strains*

| Rat strain | Authors | Tumour incidence | Maligant tumour incidence | Preponderantly tumours | General comments |
|---|---|---|---|---|---|
| ACJ/N | A. Maekawa & S. Odashima (1975) | 139/264 | 49/264 | testis, pituitary gland, uterus | old rats, wide range of tumours |
| SD | H. J. Kinkel (1971) | 59/350 | 4/350 | — | old rats |
| SD1 | W. F. McKenzie & | 147/258 | 21/258 | skin | wide range of |
| SD2 | F. M. Garner (1973) | 103/268 | 11/268 | liver | tumours, differences |
| SD3 | | 189/535 | 38/535 | lymphoret. syst. | between SD strains |
| SD4 | | 80/217 | 17/217 | lymphoret. syst. | of the individual laboratories |
| Osborne | W. F. McKenzie & | 71/131 | 17/131 | lymphoret. syst. | wide range of |
| Oregon | F. M. Garner (1973) | 157/673 | 27/673 | lymphoret. syst. | tumours |
| Wistar germfree | M. Pollard & M. Kajima (1970) | 5/33 | 0/33 | | |
| F344 | B. Sass *et al.* (1975) | 517/352 | 160/352 | lymphoret. syst., mamma, pituitary gland, testis | wide range of tumours |
| F344 germfree | M. R. Sacksteder (1976) | 98/180 | 97/180 | lymphoret. syst., mamma | fewer tumours in germfree rats |
| Brown Norway | J. D. Burek & C. F. Hollander (1977) | 394/310 | 182/310 | uterus, bladder, pituitary gland | old rats, wide range of t., peak inc. of some tumours |
| BDX | Zöller *et al.* (1977) (this paper) | 60/97 | 53/97 | connective tissue, skin, gastrointestinal, genitourinary, mamma | old rats, wide range of tumours |

in different organs was observed, but there were marked differences between the various rat strains. The BDX rats showed a high frequency of sarcomas of the connective tissue and of tumours of the gastrointestinal tract.

Whereas the general tumour incidence in BDX rats was comparable with that in other strains, the incidence of spontaneous malignant tumours seemed to be exceptionally high, only Sass *et al.* (1975) describing a similar frequency in F344 rats. In our study, the high incidence of malignant tumours may be partly explained by the fact that, in addition to histological classification, malignancy was also verified by the transplantability criterion. This was not the case in the other studies listed in Table III.

From 41 s.c.-implanted tumours, until now 34 (83%) have been retransplanted. Since some of the primary tumour implants took as long as 12 months to grow, it is to be expected that the percentage of tumours growing as s.c. implants will even increase. The latent period in the first passage was 4 weeks for undifferentiated tumours and as long as 12 months for well-differentiated tumours, which in some instances could not be differentiated histologically from benign tumours. As suggested by Knox, Linder and Friedell (1970) there might exist some relationship between the latent period and the malignancy of the primary tumour. With the exception of mammary adenocarcinomas, the latent period decreased after the 4th and 5th passage to 2–6 weeks. The pretreatment of infected primary tumours with 0·1% $Hg(CN)_2$ . HgO proved to be effective; only in one instance was a line lost because of infection of the first implant.

According to our findings the BDX rat strain seems to be ideal for many kinds of studies of autochthonous tumours as well as for the establishment of spontaneous tumour lines for immunological experiments. This is concluded from the high incidence and the unusually wide spectrum, with respect to organ location and degree of differentiation, of spontaneous malignant tumours.

We are indebted to Dr Wahrendorf, German Cancer Research Centre, for performing the life-table analysis.

### REFERENCES

BALDWIN, R. W. (1973) Immunological Aspects of Chemical Carcinogensis. *Adv. Cancer Res.*, **18**, 1.

BALDWIN, R. W. & EMBLETON, M. J. (1969) Immunology of Spontaneously Arising Mammary Adenocarcinomas. *Int. J. Cancer*, **4**, 430.

BUREK, J. D. & HOLLANDER, C. F. (1977) Incidence Patterns of Tumours in BN/Bi Rats. *J. natn. Cancer Inst.*, **58**, 99.

DRUCKREY, H., IVANKOVIC, S. & SCHMÄHL, D. (1967) Organotrope Carcinogene Wirkungen bei 65 verschiedenen N-Nitroso-Verbindungen an BD-Ratten. *Z. Krebsforsch.*, **69**, 102.

DRUCKREY, H. (1971) Genotypes and Phenotypes of Ten Inbred Strains of BD Rats. *Arzneimittel Forsch.*, **21**, 1274.

KANN, J., SPIEGELHALDER, B., EISENBRAND, G. & PREUSSMANN, R. (1978) Occurrence of Volatile N-Nitrosoamines in Animal Diets. *Z. Krebsforsch.* (in press).

KINKEL, H. J. (1971) Spontantumoren bei Sprague-Dawley-Ratten. *Z. Versuchstierk.*, **13**, 97.

KNOX, W. E., LINDER, M. & FRIEDELL, G. H. (1970) A Series of Transplantable Rat Mammary Tumours with Graded Differentiation, Growth Rate and Glutaminase Content. *Cancer Res.*, **30**, 283.

LAMON, E. W. (1974) The Immune Response to Virally Determined Tumor-associated Antigens. *Biochim. biophys. Acta*, **355**, 149.

McKENZIE, W. F. & GARNER, F. M. (1973) Comparison of Neoplasms in Six Sources of Rats. *J. natn. Cancer Inst.*, **50**, 1243.

MAEKAWA, A. & ODASHIMA, S. (1975) Spontaneous Tumours in ACI/N Rats. *J. natn. Cancer Inst.*, **55**, 1437.

MOLONEY, W. C., BOSCHETTI, A. E. & KING, V. P. (1970) Spontaneous Leukemia in Fischer Rats. *Cancer Res.*, **30**, 41.

NOBLE, R. L., HOCHACHKA, B. C. & KING, D. (1975) Spontaneous and Estrogen-induced Tumors in Nb Rats and their Behaviour after Transplantation. *Cancer Res.*, **35**, 766.

POLLARD, M. & KAJIMA, M. (1970) Lesions in Aged Germfree Wistar Rats. *Am. J. Path.*, **61**, 25.

SACHS, R. (1959) Life Table Technique in the Analysis of Response-time Data from Laboratory Experiments on Animals. *Toxicol. appl. Pharmacol.*, **1**, 203.

SACKSTEDER, M. R. (1976) Occurrence of Spontaneous Tumors in the Germfree F344 Rat. *J. natn. Cancer Inst.*, **57**, 1371.

SASS, B., RABSTEIN, L. S., MADISON, R., NIMS, R. M., PETERS, R. L. & KELLOFF, G. J. (1975) Incidence of Spontaneous Neoplasms in F344 Rats Throughout the Natural Life-span. *J. natn. Cancer Inst.*, **54**, 1449.